# Anti-fibrotic Treatment for Pulmonary Fibrosis Induced by COVID-19: A Case Presentation

**DOI:** 10.5152/TJAR.2021.20450

**Published:** 2022-06-01

**Authors:** Bahar Sakızcı Uyar, Kerem Ensarioğlu, E. Bahar Kurt, Derya Özkan, Serra Özbal Güneş

**Affiliations:** 1 Department of Anaesthesiology and Reanimation, University of Health Sciences, Dışkapı Yıldırım Beyazıt Training and Research Hospital, Ankara, Turkey; 2 Department of Pulmonary Medicine, University of Health Sciences, Dışkapı Yıldırım Beyazıt Training and Research Hospital, Ankara, Turkey; 3 Department of Radiology, University of Health Sciences, Dışkapı Yıldırım Beyazıt Training and Research Hospital, Ankara, Turkey

**Keywords:** Antifibrotic, COVID-19, pirfenidone, pulmonary fibrosis, respiratory distress

## Abstract

Coronavirus disease 19 infection clinical presentation varies from asymptomatic cases to acute respiratory distress syndromes. In some cases, pulmonary fibrosis is observed after or during the disease. Pirfenidone is an agent approved for the treatment of idiopathic pulmonary fibrosis. Here we report a patient treated with pirfenidone for pulmonary fibrosis related to coronavirus disease 19.

## Introduction

Coronavirus disease 19 (COVID-19) infection, originated from Wuhan, China, was declared a pandemic on March 11, 2020. To date, there are about 65.3 million infected people and about 1.5 million deaths worldwide.^1^ Coronavirus disease 19 infection may be completely asymptomatic, but in some cases, it also may cause systemic hyperinflammation, pulmonary fibrosis, and multi-organ failure. Available treatments currently focus on reducing the severity of the infection and preventing complications.^2^ It is thought that antifibrotics may be effective in the prevention and treatment of fibrosis caused by COVID-19.^3,4^

Pirfenidone is an anti-fibrotic agent approved for the treatment of idiopathic pulmonary fibrosis (IPF). It has been shown that pirfenidone has anti-inflammatory and anti-fibrotic effect by reducing the accumulation of inflammatory cells and the proliferation of fibroblasts.^5^ In this case report, the use of pirfenidone in a patient who developed fibrosis due to COVID-19 will be presented. Consent from the patient and approval from the Republic of Turkey Ministry of Health were obtained for this case report (Approval ID: UYAR-2020-12-18T12_44_18).

## Case Presentation

**Figure 1.**
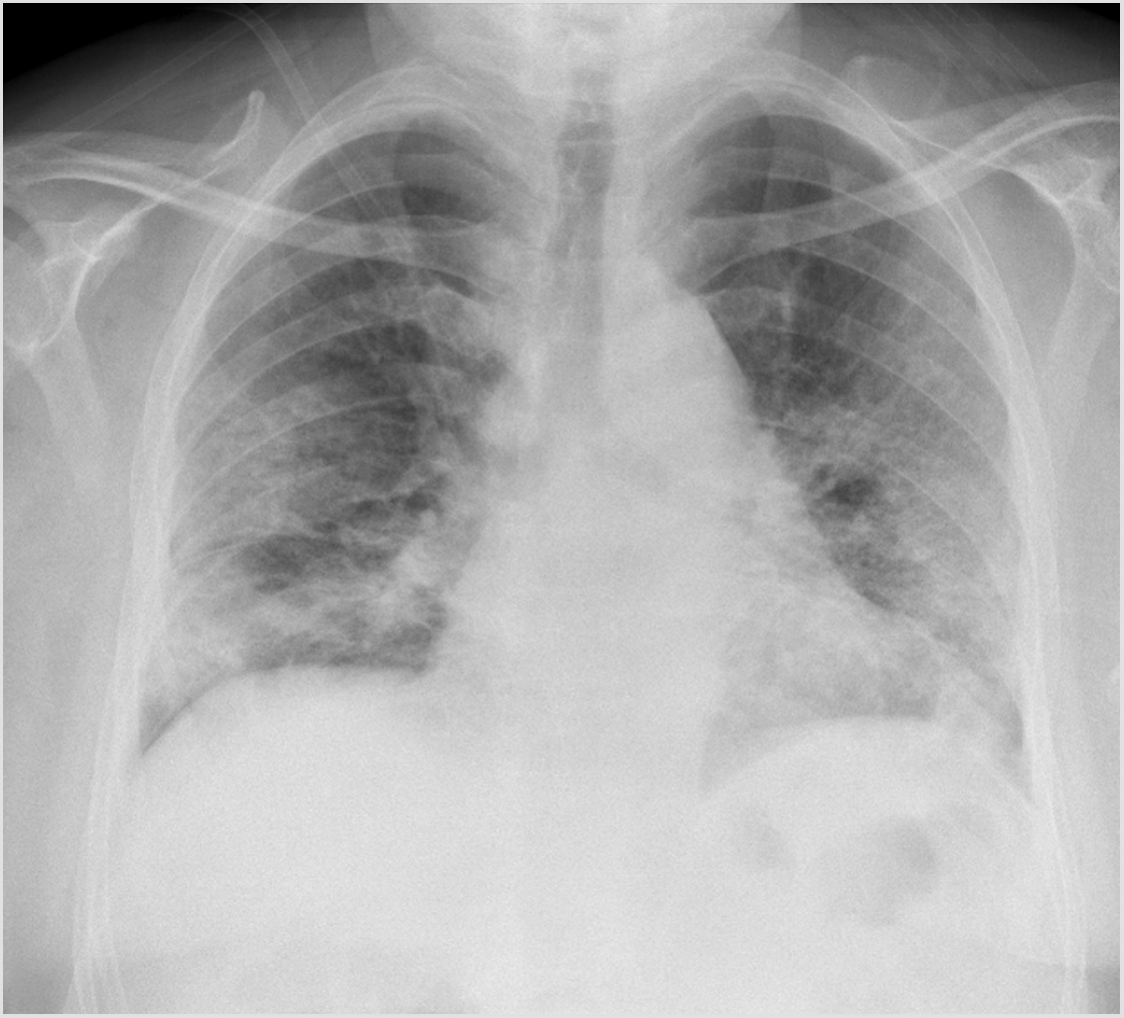
Bilateral opacities predominantly on lower peripheral zones.

A 64-year-old male with known hypertension and chronic obstructive pulmonary disease (COPD) presented to the emergency department with fever and cough. The patient did not require long-term oxygen therapy and was under inhaler treatment for COPD. Bilateral opacities predominantly on lower peripheral zones were observed on posteroanterior chest X-ray (Figure 1). Reverse transcription-polymerase chain reaction testing for severe acute respiratory syndrome coronavirus 2 (SARS-CoV-2) RNA was found to be tested negative; however, the patient was found to be tested positive for COVID-19 rapid antibody test which evaluates the presence of any antibody. The patient was then hospitalized with the diagnosis of SARS-COV-2 pneumonia due to clinical, radiological, and rapid antibody test positivity. The patient’s vitals were within normal ranges, excluding oxygen saturation (SpO_2_) which required oxygen therapy with a diffuser mask. Initial treatment included favipiravir, intravenous dexamethasone 6 mg, and low-molecular-weight heparin. Elevation of inflammatory markers was observed on the fourth day of treatment, and piperacillin–tazobactam was initiated as empirical therapy. Within 2 days, the patient’s general status deteriorated. Oxygen saturation was 80% despite 10 L min-1 oxygen therapy and bilateral diffuse infiltration was observed in the follow-up chest X-ray.

The patient was transferred to the intensive care unit (ICU) and a high-flow nasal cannula (HFNC) at 60 L min-1 with 100% fractional inspired oxygen (FiO_2_) was initiated along with an intermittent continuous positive airway pressure support. Due to an increase in ferritin, D-dimer, and C-reactive protein levels, tocilizumab at a total dosage of 800 mg (8 mg kg-1) was administered by 2 consecutive intravenous infusions 48 hours apart for a possible cytokine release syndrome. Treatment response was observed with limited improvement in hypoxia and reduction in opacities in a chest X-ray. Despite treatments, SpO_2_ > 90% could be achieved with HFNC at 40 L min-1 and 80% FiO_2_ during ICU follow-up.

**Figure 2.**
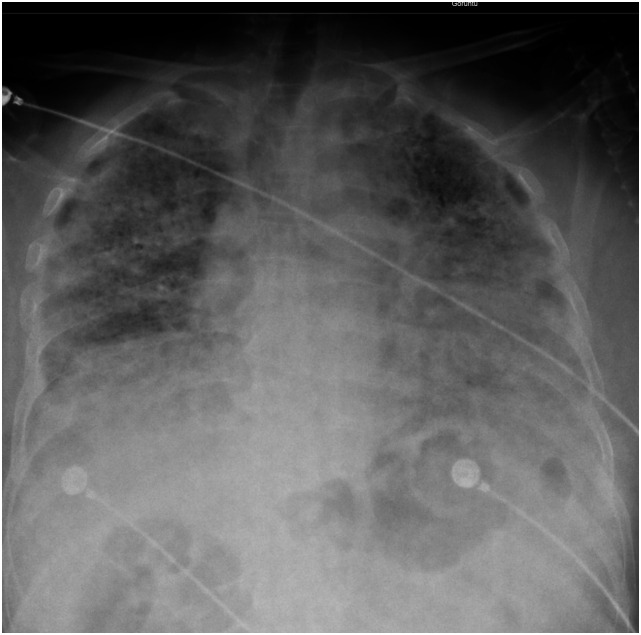
Diffuse pattern consistent with interstitial pulmonary disease.

Thirty days after the initial diagnosis of COVID-19, the patient’s general condition once again deteriorated in ICU. Respiratory distress was present and SpO_2_ was 85% despite HFNC at 60 L min-1 and 100% FiO_2_. Hypoxia and normocarbia were present in arterial blood gas analysis. Due to the suspicion of fibrosis in the requested chest X-ray, high-resolution chest computed tomography (HRCT) was performed, and diffuse pulmonary fibrosis was confirmed (Figures 2–3). Methyl-prednisolone 1000 mg IV was administered for 3 days. Since hypoxia did not improve, the patient was presented to the council of pulmonary medicine and anaesthesiology and reanimation departments. Pirfenidone treatment was started after obtaining patient consent with the decision of the council. An initial pulmonary function test for diffusing capacity of the lung for carbon monoxide could not be performed due to the patient’s condition. Instead of the standardized weekly dosage increment, the 3-day protocol was preferred due to the severity of the patient. The maximum dosage of 2400 mg per day was reached within 9 days. Patient’s saturation was improved and SpO_2_ > 90% with an oxygen mask was observed to be at 6 L min-1 O_2_.

**Figure 4.**
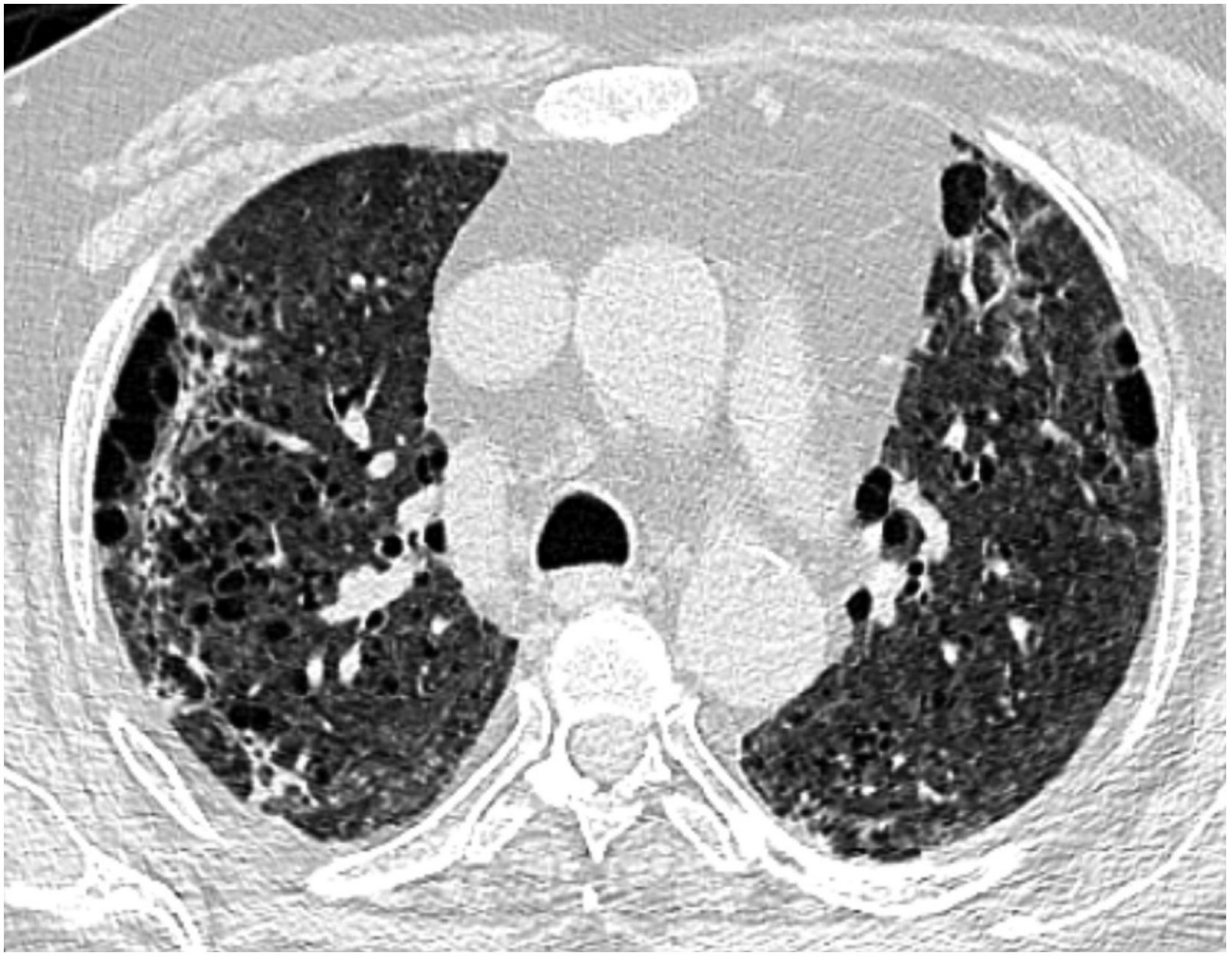
Improvement of central parenchyma while peripheral emphysema was more prominent at the first follow-up HRCT.

The patient was then admitted to the pulmonary medicine department for further treatment. Pirfenidone was continued at the maximum dosage. Inhaler treatment consisting of steroid and beta-agonist was initiated and steroid treatment was continued orally. Upon confirmation of fibrosis resolution at chest X-ray, the patient was discharged from the ward under pirfenidone, steroid, inhaler therapy, and long-term oxygen therapy with the nasal cannula at 2-4 L min-1. The patient was re-evaluated at the outpatient setting after a month following discharge. Radiological and clinical improvement was observed. Steroid treatment was stopped and a total of 90 days for pirfenidone treatment were planned. Response to treatment was seen in HRCT evaluation at the first and second month of evaluation (Figures 4–).

## Discussion

Coronavirus disease 19 infection can be divided into 3 main stages. Patients are often asymptomatic or have fatigue, cold, and sore throat in the first initial stage. The second stage consists of the acute inflammatory phase, which is followed by the recovery stage. In some patients, pulmonary healing is complicated by abnormal immune response and resolution occurs by pulmonary fibrosis. Control of these stages with immunomodulatory and anti-inflammatory treatment is essential to prevent further complications.^3,4^ Dexamethasone of 6 mg was administered to this case in accordance with World Health Organization guidelines.^6^ Tociluzimab is considered to treat cytokine storm associated with the COVID-19 and was administered at a dose of 8 mg kg-1^7^ However, pulmonary fibrosis was observed within a month despite this treatment. Although acute exacerbation of interstitial lung disease has been reported during tocilizumab therapy for rheumatoid arthritis, exacerbation occurred after 48 weeks of initiation of tocilizumab.^8^ In addition, all of the fibrosis cases related to tocilizumab reported to the worldwide FDA Adverse Event Reporting System had rheumatologic disease.^9^ In this case, we speculated that the cause of fibrosis was COVID-19 not tocilizumab since this patient did not have a known rheumatologic disease and the time between the development of fibrosis and tocilizumab treatment was 3 weeks. Thereupon, we started pulse steroid therapy, which has been shown to be beneficial for the late phase of COVID-19 with respiratory failure.^10^ Since hypoxia did not improve, pirfenidone was initiated with the decision of the council.

Pirfenidone is an agent approved for the treatment of IPF. It downregulates anti-fibrotic processes, mainly by modifying fibroblast activity.^5^ It has been shown to be effective in the inhibition of lung injury, interleukin (IL)-1, and IL-6 activity.^11,12^ While licensed for IPF treatment, pirfenidone had been tested on other interstitial lung diseases with limited success.^13^ Studies of pirfenidone’s role in pulmonary fibrosis caused by COVID-19 are ongoing, and most available studies currently consist of case reports and commentaries. 

Common side effects of pirfenidone are often considered tolerable, which include rash, nausea, diarrhea, and headache. The main limitation would be drug-induced liver injury; hence, evaluation of liver functions is justified before the initiation of treatment and monthly during treatment.^14,15^ There is no consensus on the initiation protocol of pirfenidone or other anti-fibrotic treatments on COVID-19 fibrosis, and in most cases, a standardized approach for IPF is preferred.

## Conclusion

Pirfenidone could be used in the treatment of COVID-19-induced pulmonary fibrosis, with non-life-threatening side effects and possible beneficial effects. Protocols for initiation, continuation, and cessation of anti-fibrotic treatment have yet to be fully prepared or agreed upon. Further studies are required for the investigation of pirfenidone’s role in COVID-19-related fibrosis.

### Declaration of Interests:

The authors have no conflict of interest to declare.

## Figures and Tables

**Figure 3. f5-tjar-50-3-228:**
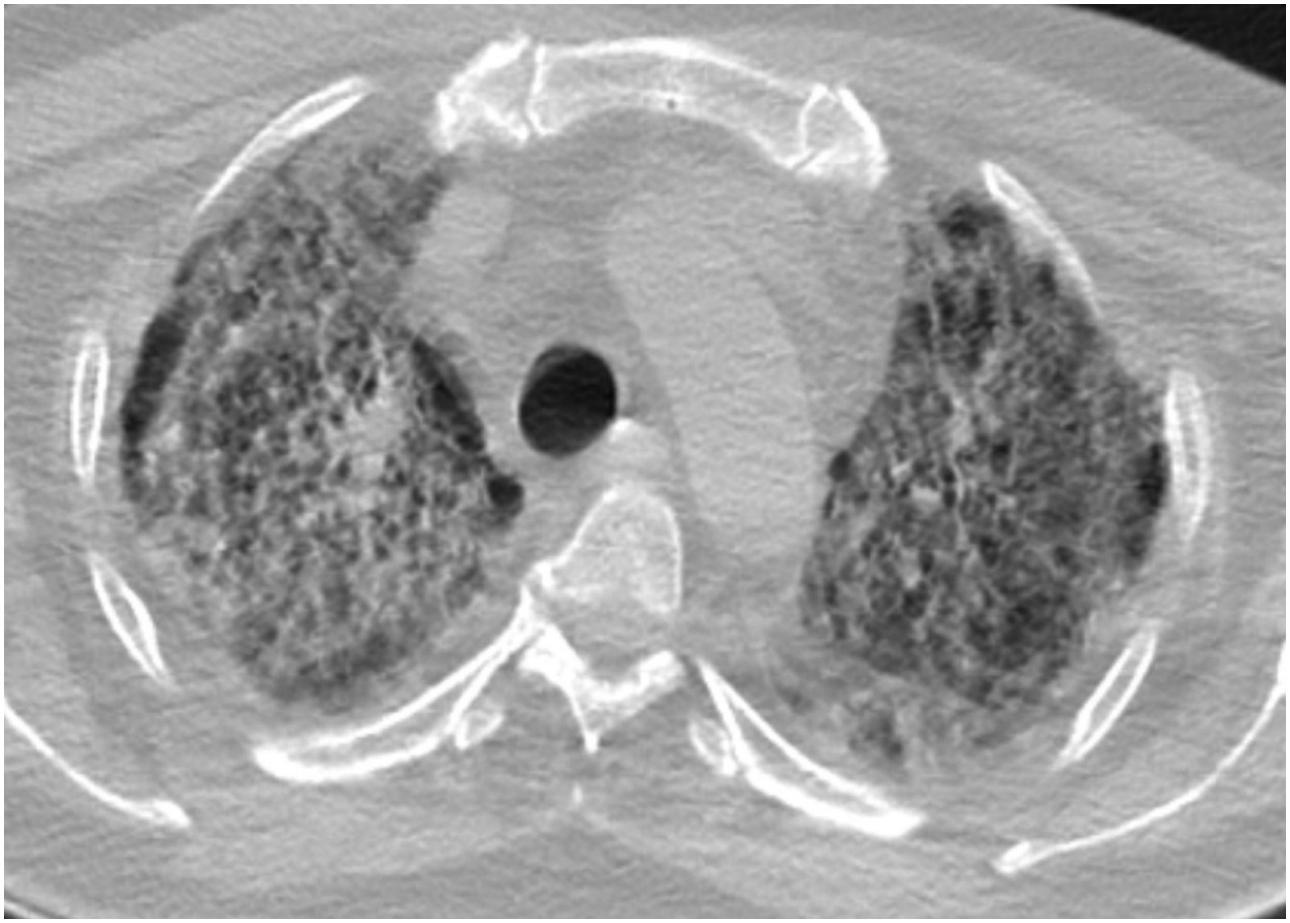
Subpleural and central emphysema along with diffuse honeycomb.

**Figure 5.**
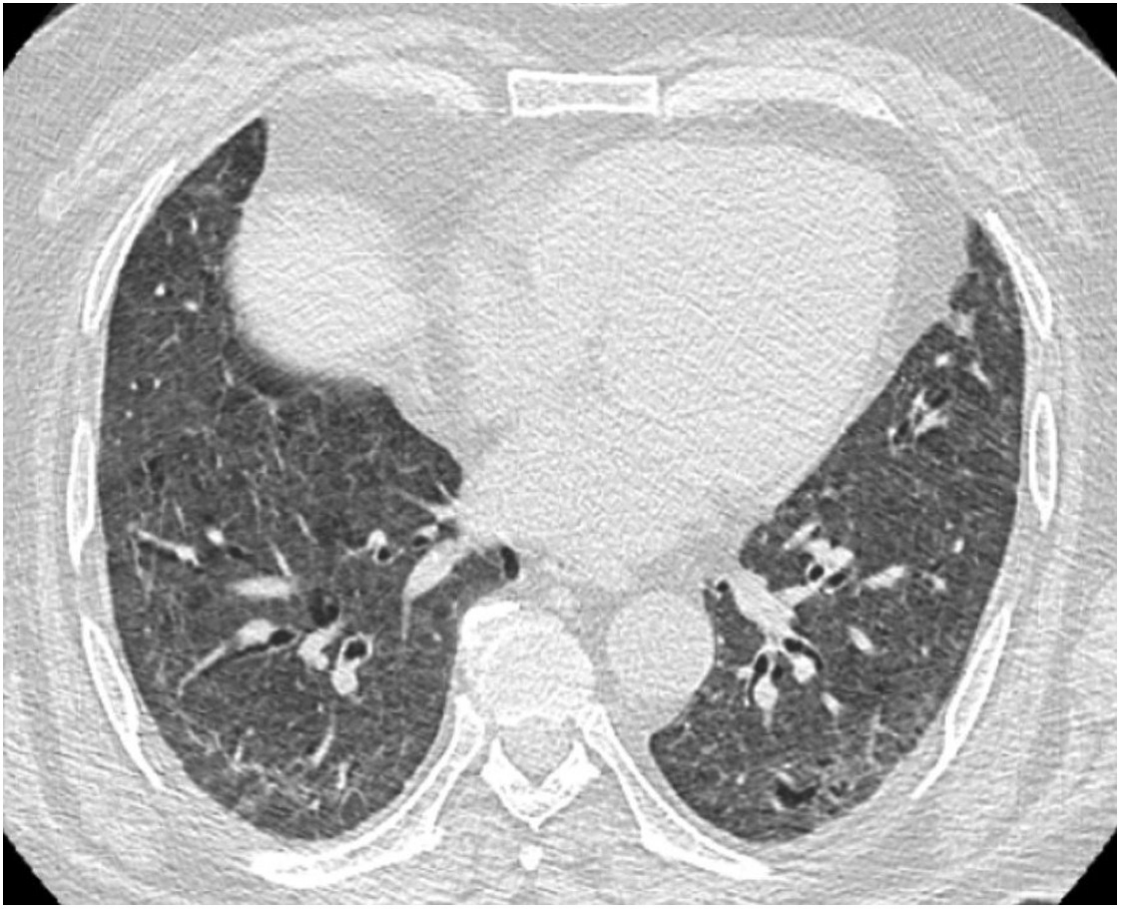
Reduction in lower lobe fibrosis at the second follow-up HRCT.
